# Tic severity and quality of life in children with tic disorders: the chain mediating effects of stigma and anxiety

**DOI:** 10.3389/fpsyt.2026.1867082

**Published:** 2026-08-24

**Authors:** Yan Wang, Fengming Zhu, Jian Zhang, Shuting Ye

**Affiliations:** Department of Pediatrics, The First People’s Hospital of Zhangjiagang City, Suzhou, Jiangsu, China

**Keywords:** anxiety, children, mediation, quality of life, stigma of disease, tic disorder

## Abstract

**Objective:**

To explore the association between tic severity and quality of life (QoL) in children with tic disorder (TD) and the hypothesized chain mediating roles of stigma and anxiety in this association.

**Methods:**

A total of 198 children with TD (January 2023–October 2025) were enrolled. Data were collected using the Yale Global Tic Severity Scale (YGTSS), Stigma Scale for Mental Illness in Children (SSMI-C), Screen for Child Anxiety Related Emotional Disorders (SCARED), Pediatric QoL Inventory 4.0 (PedsQL 4.0), and a general questionnaire. Correlation and chain mediation analyses were performed using SPSS 26.0, and mediation effects were verified using the bootstrap method (5,000 re-samples).

**Results:**

Among the 198 children with TD, the mean total scores were as follows: YGTSS, 41.19 ± 13.34; SSMI-C, 41.12 ± 9.98; SCARED, 26.29 ± 6.07; and PedsQL 4.0, 62.29 ± 14.10. PedsQL 4.0 scores differed significantly according to gender, monthly family income, and disease course (all P<0.05). Pearson correlation analysis showed that YGTSS, SSMI-C, and SCARED scores were negatively correlated with PedsQL 4.0 scores (r = -0.791, -0.743, and -0.689, respectively; all P<0.05), whereas YGTSS scores were positively correlated with SSMI-C and SCARED scores (r = 0.831 and 0.659, respectively; both P<0.05). SSMI-C scores were positively correlated with SCARED scores (r = 0.609, P<0.05). The direct effect of YGTSS on PedsQL 4.0 was significant, accounting for 53.89% of the total effect. Three significant indirect pathways were identified: (1) YGTSS→SSMI-C→PedsQL 4.0 (effect = -0.195, 23.35%); (2) YGTSS→SCARED→PedsQL 4.0 (effect = -0.142, 17.01%); and (3) YGTSS→SSMI-C→SCARED→PedsQL 4.0 (effect = -0.048, 5.75%).

**Conclusion:**

Tic severity is directly associated with the QoL in children with TD and is also indirectly associated with the QoL through the individual mediating effects of stigma and anxiety, as well as the hypothesized chain mediating pathway of stigma and anxiety. These cross-sectional findings are consistent with a hypothesized sequential pathway and suggest that interventions targeting stigma and anxiety-including cognitive-behavioral therapy, peer-support programs, school-based psychoeducation, and family interventions-should be evaluated as potential strategies to improve QoL in children with TD.

## 1 Introduction

Tic disorders (TD) are a group of neurodevelopmental disorders with onset typically occurring in childhood or adolescence. They are characterized by involuntary, repetitive, and rapid motor and/or vocal tics affecting one or more body parts and is often accompanied by a variety of psychological and behavioral comorbidities (1). According to relevant statistics, the prevalence of TD among children and adolescents ranges from 2.4% to 3.3%, with males being affected at a significantly higher rate than females (2).

As the disease progresses, tic symptoms not only affect children’s attention, learning ability, and social interactions but are also associated with a significant decline in the quality of life (QoL). As an important indicator of children’s health status, QoL encompasses multiple dimensions, including physical, psychological, and social functioning (WHOQOL-BREF). Previous studies have shown that tic severity is a key factor associated with QoL in children, with more severe symptoms being associated with greater impairment in QoL (3). However, the specific psychological mechanisms underlying the association between tic severity and QoL have not been fully elucidated. In particular, empirical research examining sequential pathways involving symptom-related psychosocial factors and emotional disorders remains limited.

Specifically, although previous studies have separately reported associations between tic severity and stigma, stigma and anxiety, and anxiety and QoL, no study has examined whether stigma and anxiety function as sequential mediators within a single model among children with TD. The present study aims to fill this gap by empirically examining the hypothesized chain pathway (Tic severity → stigma → anxiety → QoL) using a cross-sectional design while acknowledging the causal limitations of this approach.

Stigma refers to feelings of shame, discrimination, and self-stigmatization resulting from an individual’s disease characteristics and is a common psychological problem among children with chronic illnesses (4). The involuntary tic symptoms experienced by children with TD are highly visible and may lead to ridicule, rejection, and misunderstanding from peers, thereby contributing to a sense of stigma, social avoidance, self-denial, and related behaviors (5). Stigma has been identified as one of several important risk factors for anxiety in children with TD, alongside neurobiological vulnerabilities, genetic predisposition, and other psychosocial stressors. The prevalence of comorbid anxiety disorders among children with TD ranges from 30% to 50%, reflecting the multifaceted etiology of anxiety in this population (6). A recent meta-analysis reported a pooled prevalence of 34.1% for any anxiety disorder in youth with TD, with social phobia and generalized anxiety disorder being the most common subtypes (7). Anxiety may not only exacerbate tic symptoms but also further impair children’s daily functioning and QoL. Importantly, while stigma and anxiety are interrelated, stigma represents one pathway among multiple mechanisms through which tic symptoms may influence emotional outcomes, and it should not be construed as the primary or sole driver of anxiety in children with TD.

Accordingly, the present study proposes the following hypothesis, tested within a cross-sectional framework: tic severity is associated with QoL both directly and indirectly through three hypothesized pathways: the independent mediating effects of stigma and anxiety, and the chain mediating effect of stigma leading to anxiety. We explicitly acknowledge that the proposed ordering of stigma and anxiety is theory-driven and cannot be empirically verified using the present cross-sectional data. Furthermore, while our model positions stigma as a potential precursor to anxiety, we recognize that this represents only one plausible pathway among several. Anxiety in children with TD has diverse and multifactorial origins, and our focus on the stigma→anxiety pathway is not intended to minimize the importance of other biological, genetic, or psychosocial contributors. Rather, we aim to examine whether this specific pathway is statistically consistent with the data, while acknowledging that anxiety can arise through mechanisms entirely independent of stigma experiences. By examining these relationships, this study seeks to identify a theory-informed statistical pathway that, if supported, may guide future longitudinal and intervention research.

## 2 Data and methods

### 2.1 Research objects

This study was approved by the Ethics Committee of the First People’s Hospital of Zhangjiagang City. Written informed consent was obtained from the legal guardians of all the participating children. The sample consisted of 198 pediatric patients with TD who received care at our facility between January 2023 and October 2025. Eligibility for inclusion was based on the following criteria: (1) meeting the diagnostic criteria for TD according to the *Chinese Expert Consensus on Diagnosis and Treatment of Tic Disorders in Children (2017 Practical Edition)* (8); (2) aged 6–16 years, with basic reading and writing abilities, language comprehension and expression skills, and the ability to complete the self-assessment scales independently or under the standardized neutral guidance of researchers; and (3) having legal guardians who were informed about the study and provided written informed consent. The exclusion criteria were as follows: (1) comorbid autism spectrum disorder, moderate-to-severe intellectual disability, epilepsy, cerebral palsy, other neurodevelopmental disorders, or organic diseases of the nervous system; (2) severe psychiatric disorders such as schizophrenia or bipolar disorder; (3) major negative life events within the previous three months (e.g., bereavement or major family changes); and (4) receiving systematic psychotherapy or high-dose psychotropic medications that could interfere with the completion of emotion-related assessments.

Diagnoses were made independently by two pediatric psychiatrists (attending physician level or higher). In cases of disagreement, a third senior psychiatrist made the final diagnostic decision. No formal inter-rater reliability analysis was conducted for the diagnoses; however, a consensus was reached in all cases following discussion.

The sample size was estimated using G*Power 3.1 software based on a chain mediation model. The significance level was set at α = 0.05, statistical power at 1 − β = 0.90, and effect size at f² = 0.15 (medium). Considering the number of independent, mediating, and dependent variables included in the study, the minimum required sample size was estimated to be 180. Participants were recruited consecutively from January 2023 to October 2025. Of 235 eligible children with TD, 212 agreed to participate (90.2% participation rate); 198 provided valid questionnaires after excluding 14 with incomplete data. The final sample therefore represents a consecutive clinical series from a single tertiary hospital, limiting generalizability to community or non-referred populations.

### 2.2 Data collection instruments

#### 2.2.1 Participant information sheet

A researcher-designed questionnaire was used to collect participants’ demographic and clinical information, including sex, age, place of residence, monthly family income, and disease duration.

#### 2.2.2 Yale global tic severity scale

Tic severity was assessed using the Yale Global Tic Severity Scale (YGTSS) (9). The scale evaluates tic severity across two domains: motor and phonic tics. Each domain is rated on five dimensions: number, frequency, intensity, complexity, and interference, with each dimension scored from 0–5. The total tic severity score ranges from 0 to 100 and is calculated as the sum of the motor and phonic tic severity scores (each ranging from 0 to 50). An additional impairment rating (0–50) was collected but not included in the total tic severity score used in this study. The Cronbach’s α coefficient of the YGTSS in this study was 0.862.

#### 2.2.3 Social stigma scale for mental illness—Chinese version

The SSMI-C (10) is a Chinese version of the Stigma Scale for Mental Illness adapted for children and adolescents with Tourette syndrome. It has been validated in a Chinese pediatric population aged 6–16 years with TD (Cronbach’s α = 0.82–0.89; test–retest reliability r = 0.85). The scale comprises four dimensions: perceived discrimination, shame experience, social avoidance, and self-denial, comprising 22 items. Specifically, the perceived discrimination dimension included six items, the shame experience dimension 5 items, the social avoidance dimension 6 items, and the self-denial dimension 5 items. Each item was rated on a 4-point Likert scale (1 = completely inconsistent, 2 = occasionally consistent, 3 = frequently consistent, and 4 = completely consistent), with three items scored in reverse. Total scores range from 22 to 88, with higher scores indicating greater stigma associated with tic symptoms. In the present study, Cronbach’s coefficient for the overall scale was 0.843, while the coefficients for the individual dimensions ranged from 0.772 to 0.835, indicating a good reliability.

#### 2.2.4 Screen for child anxiety related emotional disorders

The SCARED is a widely used instrument for assessing anxiety symptoms in children and adolescents (11). It is applicable to individuals aged 6–16 years and includes five dimensions: somatic/panic symptoms, generalized anxiety, separation anxiety, social phobia, and school phobia, comprising 41 items. Items are scored on a 3-point scale (0 = not true or hardly ever true, 1 = somewhat true or sometimes true, and 2 = very true or often true), yielding a total score ranging from 0 to 82. Higher scores indicate more severe anxiety symptoms, and a total score of 23 or more suggests clinically significant anxiety. In this study, the scale demonstrated good internal consistency, with a Cronbach’s α coefficient of 0.895.

#### 2.2.5 Pediatric QoL inventory 4.0

The Pediatric QoLInventory 4.0 Generic Core Scales (PedsQL 4.0; child self-report version) (12) was used to assess the QoL. For children aged 6–8 years, the questionnaire items were read aloud by trained research staff using a standardized neutral tone. Age-specific versions (5–7, 8–12, and 13–18 years) were administered according to the instrument manual. The scale is a widely used generic measure of pediatric QoL and includes four dimensions: physical, emotional, social, and school functioning, comprising a total of 23 items. Each item is rated on a 5-point scale (0 = never, 4 = almost always), with responses reverse scored and linearly transformed to a 0–100 scale. The total score was calculated as the average score across all dimensions, with higher scores indicating better QoL. In the present study, Cronbach’s α coefficient of the scale was 0.901, indicating good reliability and validity.

### 2.3 Data collection

The YGTSS was administered by two trained psychological therapists who had completed a standardized workshop on tic disorder assessments. The inter-rater reliability for the YGTSS total score was good (ICC = 0.89, 95% CI: 0.84–0.93), based on 20 randomly selected cases.

Once the eligibility criteria were met, both the participating children and their legal guardians received a detailed explanation of the study’s purpose, procedures, and relevant considerations. After written informed consent was obtained, the participants completed the self-administered questionnaires in a designated quiet room. For younger children, the research staff read each item aloud using standardized neutral wording to ensure comprehension while avoiding leading prompts.

Upon completion, all questionnaires were immediately reviewed on-site for completeness and consistency. Questionnaires with missing data or patterned response sets were excluded from the analysis. A total of 212 eligible children were approached, and 198 provided valid questionnaires for the final analysis.

### 2.4 Statistical methods

The collected data were processed and analyzed using the SPSS statistical software (version 26.0). The normality of continuous variables was assessed using the Shapiro–Wilk test. Data conforming to a normal distribution are presented as mean ± standard deviation (x̄ ± s). An independent-samples t-test was used for comparisons between the two groups. For comparisons among multiple groups, one-way analysis of variance (ANOVA) was performed, followed by Bonferroni *post hoc* tests for pairwise comparisons of means. Categorical variables are presented as n (%).

Pearson correlation analysis was conducted after confirming approximate normality (Shapiro–Wilk P > 0.05 for all variables) and linearity through visual inspection of scatter plots. Mediation analysis was performed using the PROCESS macro (version 4.2, Model 6) for SPSS, with 5,000 bootstrap resamples and bias-corrected 95% confidence intervals. Unstandardized regression coefficients (β) are reported. The following covariates were included in all mediation models: gender, age (continuous), monthly family income (categorical), and disease duration (≤1 year vs. >1 year). Multicollinearity was assessed using the variance inflation factor (VIF). R² values are reported for each mediator and outcome model. A two-sided P value < 0.05 was considered statistically significant.

## 3 Results

### 3.1 Characteristics of the study participants

A total of 198 children with TD were included in this study. Among them, 138 (69.70%) were male and 60 (30.30%) were female patients. The mean age was 9.58 ± 2.41 years (range, 6–16 years). Regarding place of residence, 112 participants (56.57%) lived in urban areas, and 86 (43.43%) lived in rural areas. Monthly family income was <5,000 yuan for 38 participants (19.19%), 5,000–10,000 yuan for 86 participants (43.43%), and >10,000 yuan for 74 participants (37.38%). The disease duration ranged from 3 months to 8 years, with a mean duration of 1.92 ± 1.36 years.

The TD subtypes included Tourette disorder (n = 112, 56.57%), persistent motor or vocal tic disorder (n = 58, 29.29%), and provisional tic disorders (n = 28, 14.14%). Thirty-six children (18.18%) received pharmacotherapy (e.g., clonidine or aripiprazole). The comorbidities included attention-deficit/hyperactivity disorder (ADHD; n = 42, 21.21%), obsessive-compulsive disorder (OCD; n = 23, 11.62%), and depressive symptoms (n = 31, 15.66%), based on parent-reported clinical diagnoses or screening scores exceeding established cutoffs.

### 3.2 Scores of study scales

Among the 198 children with TD, the mean total YGTSS score was 41.19 ± 13.34, mean SSMI-C score was 41.12 ± 9.98, mean SCARED score was 26.29 ± 6.07, and mean PedsQL 4.0 score was 62.29 ± 14.10. The details are presented in **Table 1**.

**Table 1:** Score of each scale (x̄ ± s).

Item	Dimension	Score
YGTSS	Total score	41.19 ± 13.34
SSMI-C	Total score	41.12 ± 9.98
SCARED	Total score	26.29 ± 6.07
PedsQL4.0	Total score	62.29 ± 14.10

### 3.3 Comparison of PedsQL 4.0 scores among children with TD according to demographic and disease characteristics

PedsQL 4.0 scores differed significantly according to sex, monthly family income, and disease duration among children with TD (all P < 0.05). The details are presented in **Table 2**.

**Table 2:** Comparison of pedsql4.0 scores in children with TD with different demographic and disease characteristics (x̄ ± s).

Characteristic	Grouping	Number of cases	PedsQL4.0	*t/F*	*P*
Gender	Male	138	60.86 ± 14.99	2.180	0.030
	Female	60	65.56 ± 11.17		
Age (years)	6-9	114	62.71 ± 12.43	0.503	0.615
	10-16	84	61.69 ± 16.11		
Residence	Town	112	63.52 ± 11.40	1.419	0.157
	Countryside	86	60.66 ± 16.89		
Monthly family income (yuan)	<5000	38	57.41 ± 7.71	5.083	0.007
	5000-10000	86	61.32 ± 15.69^*^		
	>10000	74	65.90 ± 13.99^*#^		
Course of disease (years)	≤1	72	66.56 ± 14.57	3.313	0.001
	>1	126	59.84 ± 13.24		

^*^ *P* < 0.05 compared to the <5,000-yuan group; ^#^*P* < 0.05 compared to the 5,000–10,000 yuan group.

### 3.4 Correlation analysis between variables

**Figure 1:**
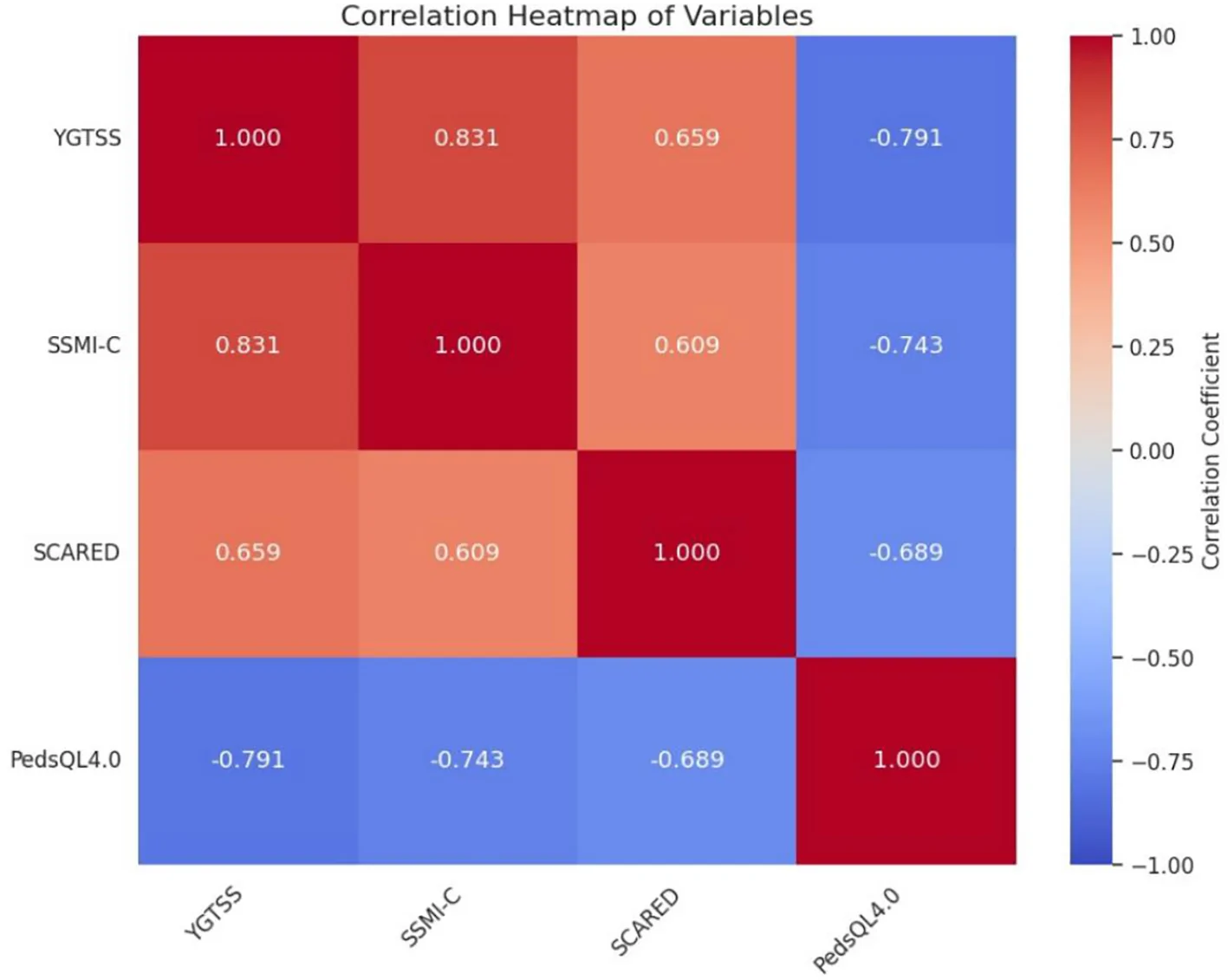
Correlation analysis between variables.

Pearson’s correlation analysis showed that the YGTSS, SSMI-C, and SCARED scores were negatively correlated with the PedsQL 4.0 scores (r = -0.791, -0.743, and -0.689, respectively; all P < 0.05). In addition, YGTSS scores were positively correlated with SSMI-C and SCARED scores (r = 0.831 and 0.659, respectively; both P < 0.05), and SSMI-C scores were positively correlated with SCARED scores (r = 0.609, P < 0.05). The details are presented in **Table 3** and **Figure 1**.

**Table 3:** Correlation analysis between variables.

Variable	1	2	3	4
1.YGTSS	1	0.831	0.659	-0.791
2.SSMI-C	0.831	1	0.609	-0.743
3.SCARED	0.659	0.609	1	-0.689
4.PedsQL4.0	-0.791	-0.743	-0.689	1

Pearson correlation analysis, all *P* < 0.05.

### 3.5 Chain mediation model of stigma and anxiety in the association between tic severity and QoL in children with TD

A chain mediation model was constructed using YGTSS as the independent variable (X), PedsQL 4.0 as the dependent variable (Y), and SSMI-C and SCARED as the mediating variables (M1 and M2). The results showed that YGTSS negatively predicted PedsQL 4.0 scores and positively predicted SSMI-C and SCARED scores. SSMI-C scores negatively predicted PedsQL 4.0 scores and positively predicted SCARED scores, while SCARED scores negatively predicted PedsQL 4.0 scores. The details are presented in **Table 4**.

**Table 4:** Chain mediation model of stigma and anxiety in tic severity and QoL in children with TD.

Outcome variable	Predictive variable	Model coefficient			
		*β*	*SE*	*t*	*P*
PedsQL 4.0	YGTSS	-0.450	0.081	-5.558	<0.001
	SSMI-C	-0.313	0.103	-3.042	0.003
	SCARED	-0.633	0.125	-5.074	<0.001
SCARED	YGTSS	0.225	0.044	5.143	<0.001
	SSMI-C	0.121	0.058	2.074	0.039
SSMI-C	YGTSS	0.622	0.030	20.953	<0.001

The coefficients are unstandardized (*β*). All models were adjusted for sex, age, monthly family income, and disease course.

Collinearity diagnostics indicated that all variance inflation factor (VIF) values were below 3.0 (YGTSS: 2.14, SSMI-C: 1.98, and SCARED: 1.76), suggesting no evidence of problematic multicollinearity. The R² values were 0.612, 0.543, and 0.683 for the SSMI-C, SCARED, and PedsQL 4.0 models, respectively.

### 3.6 Chain mediating effects of stigma and anxiety in the association between tic severity and QoL in children with TD

**Figure 2:**
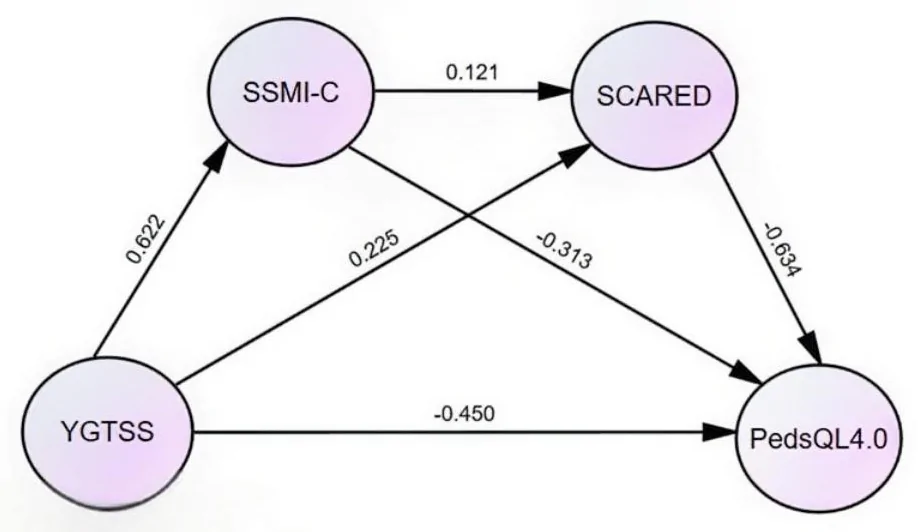
Chain mediation model of stigma and anxiety in the association between tic severity and QoL.

The direct effect of the YGTSS on PedsQL 4.0 was significant, accounting for 53.89% of the total effect. The indirect mediating effect consisted of three significant pathways: (1) YGTSS → SSMI-C → PedsQL 4.0, with a mediating effect value of -0.195, accounting for 23.35% of the total effect; (2) YGTSS → SCARED → PedsQL 4.0, with a mediating effect value of -0.142, accounting for 17.01% of the total effect; and (3) YGTSS → SSMI-C → SCARED → PedsQL 4.0, with a mediating effect value of -0.048, accounting for 5.75% of the total effect. The details are presented in **Table 5** and **Figure 2**.

**Table 5:** Chain mediation effect of stigma and anxiety on the association between tic severity and QoL.

Effect	Effect path	Effect value	*SE*	*95%CI*	Effect ratio (%)
Total effect	YGTSS-PedsQL 4.0	-0.835	0.046	-0.926~-0.744	100
Direct effect	YGTSS-PedsQL 4.0	-0.450	0.081	-0.610~-0.291	53.89
Indirect effect	YGTSS-SSMI-C-PedsQL 4.0	-0.195	0.082	-0.353~-0.037	23.35
	YGTSS-SCARED-PedsQL 4.0	-0.142	0.035	-0.221~-0.085	17.01
	YGTSS-SSMI-C-SCARED-PedsQL 4.0	-0.048	0.025	-0.100~-0.001	5.75

To examine the robustness of the hypothesized sequence, an alternative model (YGTSS → SCARED → SSMI-C → PedsQL 4.0) was tested. The indirect effect through anxiety alone was significant (effect = -0.102, SE = 0.036, 95% CI: -0.178 to -0.035), whereas the chain mediating effect from anxiety to stigma was not significant (effect = -0.012, SE = 0.014, 95% CI: -0.045 to 0.012). As a limited robustness check, the alternative sequence (tic severity → anxiety → stigma → QoL) did not yield a significant chain effect. This pattern is consistent with, but does not prove, the hypothesized ordering.

## 4 Discussion

In this theory-informed cross-sectional mediation analysis, the results showed that the mean YGTSS score among the 198 children with TD was 41.19 ± 13.34, indicating an overall moderate tic severity. This finding is consistent with the reported distribution of symptom severity among pediatric patients with TD in clinical settings in China (13), suggesting that tic symptoms were associated with noticeable impairment in daily functioning, consistent with the core characteristics of the clinical population.

The mean SSMI-C score was 41.12 ± 9.98, indicating that children with TD in this sample reported moderate levels of disease-related stigma. School-age children are at a critical stage of self-awareness and social development. Involuntary motor and vocal tics are often misunderstood by peers and teachers as disruptive or inappropriate behaviors, potentially making affected children more vulnerable to ridicule, exclusion, and social isolation. These experiences may subsequently be internalized as stigma-related perceptions, including self-denial, perceived discrimination, and social avoidance (14). It is important to note, however, that stigma is only one of multiple factors influencing the emotional well-being of children with TD, and not all affected children experience significant stigma despite having visible symptoms.

The mean SCARED score in this study was 26.29 ± 6.07, suggesting that anxiety symptoms were common among children with TD. In addition, the mean PedsQL 4.0 score was 62.29 ± 14.10, which is lower than previously reported norms for healthy Chinese children (e.g., 78.5 ± 12.3 reported by Liu et al., 2020). However, as the present study did not include a healthy control group, comparisons with external normative data should be interpreted with caution (15).

Pearson’s correlation analysis showed that the YGTSS scores were negatively correlated with the PedsQL 4.0 scores, indicating that greater tic severity was associated with poorer QoL. YGTSS scores were positively correlated with both SSMI-C and SCARED scores, suggesting that more severe tic symptoms were associated with higher levels of stigma and anxiety. As tic symptoms become more severe and visible, children may face greater risks of negative evaluation, shame, and anticipatory anxiety, potentially contributing to a vicious cycle of increasing tic severity, stigma, and anxiety (16). The SSMI-C scores were positively correlated with the SCARED scores, indicating that greater stigma was associated with more severe anxiety symptoms. Negative self-perceptions and social fears associated with stigma may function as persistent psychological stressors that contribute to anxiety in children (17). However, it is essential to interpret this association cautiously: stigma is one of multiple pathways to anxiety in children with TD, and the observed correlation does not imply that stigma is the primary or predominant cause of anxiety. Other established contributors to anxiety in this population—including neurobiological factors, genetic predisposition, comorbid ADHD or OCD, and family stress—are equally important to consider in clinical assessment and intervention. Furthermore, both stigma and anxiety were negatively correlated with QoL, suggesting that elevated levels of stigma and anxiety may impair children’s social, emotional, and school functioning, thereby reducing their overall QoL.

Consistent with previous studies, we observed that QoL was poorer in male children, those from lower-income families, and those with longer disease duration (18–20). While these factors are not the primary focus of our investigation, they represent important demographic and clinical covariates that should be considered in the assessment and management of children with TD. Future studies should systematically examine the mechanisms through which these factors influence QoL.

The present study further demonstrated that tic severity was negatively associated with QoL in children with TD, indicating that more severe tic symptoms were associated with poorer QoL. This finding is consistent with those of previous domestic and international studies (21, 22). From a physiological perspective, frequent motor and vocal tics may cause physical discomfort, such as eye strain, neck muscle soreness, and throat discomfort. In severe cases, self-injurious tics may occur, directly affecting the physical functioning. From a social perspective, overt tic symptoms can interfere with classroom learning, peer interactions, and daily activities. Some children may be misunderstood by teachers or excluded by peers because of their symptoms, resulting in impaired school and social functioning (23). Emotionally, the involuntary nature of tics—although they may be temporarily suppressed with behavioral interventions such as habit reversal training—can contribute to feelings of frustration and helplessness, further reducing QoL.

This study also found that stigma independently mediated the association between tic severity and QoL. Specifically, greater tic severity was associated with higher levels of stigma, which in turn was associated with a poorer QoL. This finding is consistent with stigma theories related to chronic diseases (24). TD is a neurodevelopmental disorder characterized by highly visible symptoms that are easily noticed by others. Children with TD are often labeled as “strange” or “naughty” and may experience ridicule, discrimination, and rejection by their peers. Prolonged exposure to such negative social experiences may lead children to internalize external negative evaluations, resulting in self-stigmatization, social withdrawal, low self-esteem, and reduced participation in social activities. These consequences may adversely affect emotional functioning and school adjustment, ultimately contributing to a poorer QoL (25).

Anxiety also independently mediated the association between tic severity and QoL, consistent with previous studies (26). Children with TD often experience tension and worry related to uncontrollable tic episodes. Anxiety may exacerbate tic symptoms, creating a reciprocal relationship between anxiety and symptom severity. Children may become excessively concerned that their symptoms will be noticed by others, particularly in school or public settings, leading to anticipatory anxiety. Persistent anxiety may contribute to sleep disturbances, impaired concentration, and social withdrawal, thereby adversely affecting physical, emotional, social, and school functioning, and reducing the overall QoL.

Importantly, stigma and anxiety demonstrated a significant chain mediating effect on the association between tic severity and QoL, accounting for 5.75% of the total effect. This finding suggests that stigma and anxiety function as independent mediators and may also operate sequentially. Specifically, the visibility and uncontrollability of tic symptoms may contribute to stigma experiences, which may subsequently increase anxiety and ultimately be associated with a reduced QoL (27). From a developmental perspective, children are in a critical stage of self-awareness and social development. Disease-related stigma may place children in a prolonged state of psychological vigilance, characterized by concerns that their symptoms will be noticed and judged by others, potentially contributing to generalized and social anxiety symptoms (28). Compared with anxiety directly related to symptoms, stigma-related anxiety may be more persistent and exert a greater negative impact on psychological and social functioning. Consequently, children may experience increased social avoidance, academic difficulties, and emotional problems, leading to further deterioration in their QoL. The identification of this potential pathway extends previous research examining the relationships among symptoms, psychological factors, emotional problems, and QoL in children with TD and may provide a useful framework for future investigations.An important consideration in the discussion of stigma is the natural course of TD. Major clinical guidelines in the United States and Europe emphasize that tic symptoms significantly decline around the end of the teenage phase for a substantial proportion of individuals, with estimates ranging from 40-60% experiencing a marked reduction or even complete remission. This favorable prognostic information is a key component of psychoeducation for families and children. Emphasizing that symptoms are likely to improve with age may itself be a powerful anti-stigma tool, helping to counter negative self-perceptions and reduce the hopelessness that can accompany chronic tic symptoms. Furthermore, the expectation of decline supports resilience and provides a developmental context that can mitigate the impact of stigma on psychological well-being. Integrating this knowledge into therapeutic interventions may help children and families maintain a more positive long-term outlook, potentially buffering the adverse effects of stigma and anxiety on quality of life.

The findings of this study have important clinical implications. Clinicians should consider incorporating routine screening for stigma and anxiety into the assessment and management of children with TD. However, prospective and interventional studies are needed to determine whether targeting these factors leads to improvements in QoL. In addition, stigma-focused interventions, including cognitive-behavioral therapy, peer-support programs, and family psychoeducation, may help reduce self-stigmatization and alleviate stigma-related distress. Finally, early identification and management of anxiety symptoms should be emphasized, particularly among children with high levels of disease-related stigma. Targeted emotional regulation interventions may help interrupt the stigma–anxiety cycle and contribute to an improved QoL.

Given that stigma is modifiable and is influenced by education, knowledge, and social attitudes, there is considerable scope for implementing prevention and neutralization strategies across multiple frameworks. At the individual level, cognitive-behavioral therapy (CBT) approaches that specifically address self-stigmatizing cognitions-such as shame, self-blame, and perceived discrimination-may help children reframe negative beliefs about themselves and their tics. Techniques including cognitive restructuring, behavioral experiments, and exposure to feared social situations can reduce stigma-related distress and social avoidance. At the peer and school level, classroom-based psychoeducation programs that explain TD and tics to classmates and teachers can reduce misunderstanding, reduce teasing and bullying, and foster a more inclusive environment. School-wide anti-stigma campaigns, similar to those implemented for mental health conditions, may help normalize TD and reduce its social burden. At the family level, parental psychoeducation and support groups can equip caregivers with accurate knowledge about TD, reduce familial stigma, and enhance their capacity to advocate for their children. Finally, at the systemic level, public health initiatives and media campaigns aimed at improving public understanding of neurodevelopmental disorders may help reduce societal stigma over the long term. Clinicians should consider referring families to available community resources and support organizations, such as patient advocacy groups, that offer peer support and educational materials. The implementation of such multi-level approaches holds promise for reducing stigma and its downstream effects on the QoL of children with TD.

## 5 Conclusion

In this cross-sectional sample, tic severity was associated with QoL both directly and indirectly through three statistically identified pathways, although causal interpretations are not supported by the study design. These pathways included the independent mediating effects of stigma and anxiety, as well as the chain mediating effect of stigma leading to anxiety. However, it is important to emphasize that anxiety in children with TD is multifactorial in origin; stigma represents one contributing pathway among multiple biological, genetic, and psychosocial mechanisms. The identification of this statistical pathway does not imply that stigma is the dominant or sole driver of anxiety in this population, and clinicians should maintain a comprehensive biopsychosocial perspective when assessing and treating children with TD. These findings suggest that, in addition to controlling tic symptoms, clinical interventions should emphasize reducing stigma and alleviating anxiety among children with TD. Targeted psychological interventions may help interrupt the symptom–stigma–anxiety pathway and improve QoL.

However, this study was a single-center cross-sectional investigation and, therefore, could only identify associations and hypothesized pathways among variables rather than establish causal relationships. In addition, the TD subtype, comorbidities (e.g., ADHD and OCD), and medication status were not included as covariates in the primary mediation model because of the limited statistical power for subgroup analyses. Although these factors were collected, the lack of adjustment for their potential effects represents an important limitation of this study.

Furthermore, although we collected data on TD subtype, comorbidity status, and medication use, the sample size was insufficient for well-powered subgroup or stratified analyses. It is plausible that the identified mediation pathways differ across TD subtypes, comorbidity profiles, and treatment groups. For example, children with TD and comorbid ADHD may have different stigma experiences than those with TD alone, given that ADHD symptoms themselves can carry social stigma. Similarly, medication status could influence tic severity and anxiety, potentially altering the mediation relationships. Importantly, because all variables were measured at the same time point, the proposed sequential pathway should be interpreted as a statistical pattern consistent with the hypothesized ordering, not as evidence of temporal or causal processes. The alternative model analysis does not resolve this limitation. Although TD subtype, comorbid ADHD/OCD/depressive symptoms, and medication status were collected, they were not included as covariates in the main mediation model because of limited statistical power for subgroup analyses. These factors are plausible confounders, and future studies with larger samples should examine whether the identified pathways differ across these subgroups. Stigma, anxiety, and QoL were all self-reported, raising the possibility of shared method variance inflating associations. Although tic severity was clinician-rated (YGTSS), common rater effects cannot be ruled out. Furthermore, the single-center consecutive clinical sample limits generalizability to community or non-referred populations. Future longitudinal studies with larger, more diverse samples are needed to verify the present findings.

## Data Availability

The original contributions presented in the study are included in the article/supplementary material. Further inquiries can be directed to the corresponding author/s.
